# Protein Splicing of Inteins: A Powerful Tool in Synthetic Biology

**DOI:** 10.3389/fbioe.2022.810180

**Published:** 2022-02-21

**Authors:** Hao Wang, Lin Wang, Baihua Zhong, Zhuojun Dai

**Affiliations:** ^1^ Materials Synthetic Biology Center, CAS Key Laboratory of Quantitative Engineering Biology, Guangdong Provincial Key Laboratory of Synthetic Genomics, Shenzhen Institute of Synthetic Biology, Shenzhen Institutes of Advanced Technology, Chinese Academy of Sciences, Shenzhen, China; ^2^ Materials Interfaces Center, Institute of Advanced Materials Science and Engineering, Shenzhen Institutes of Advanced Technology, Chinese Academy of Sciences, Shenzhen, China

**Keywords:** inteins, synthetic biology, living therapeutics, protein engineering, split inteins

## Abstract

Inteins are protein segments that are capable of enabling the ligation of flanking extein into a new protein, a process known as protein splicing. Since its discovery, inteins have become powerful biotechnological tools for applications such as protein engineering. In the last 10 years, the development in synthetic biology has further endowed inteins with enhanced functions and diverse utilizations. Here we review these efforts and discuss the future directions.

## Introduction

**FIGURE 1:**
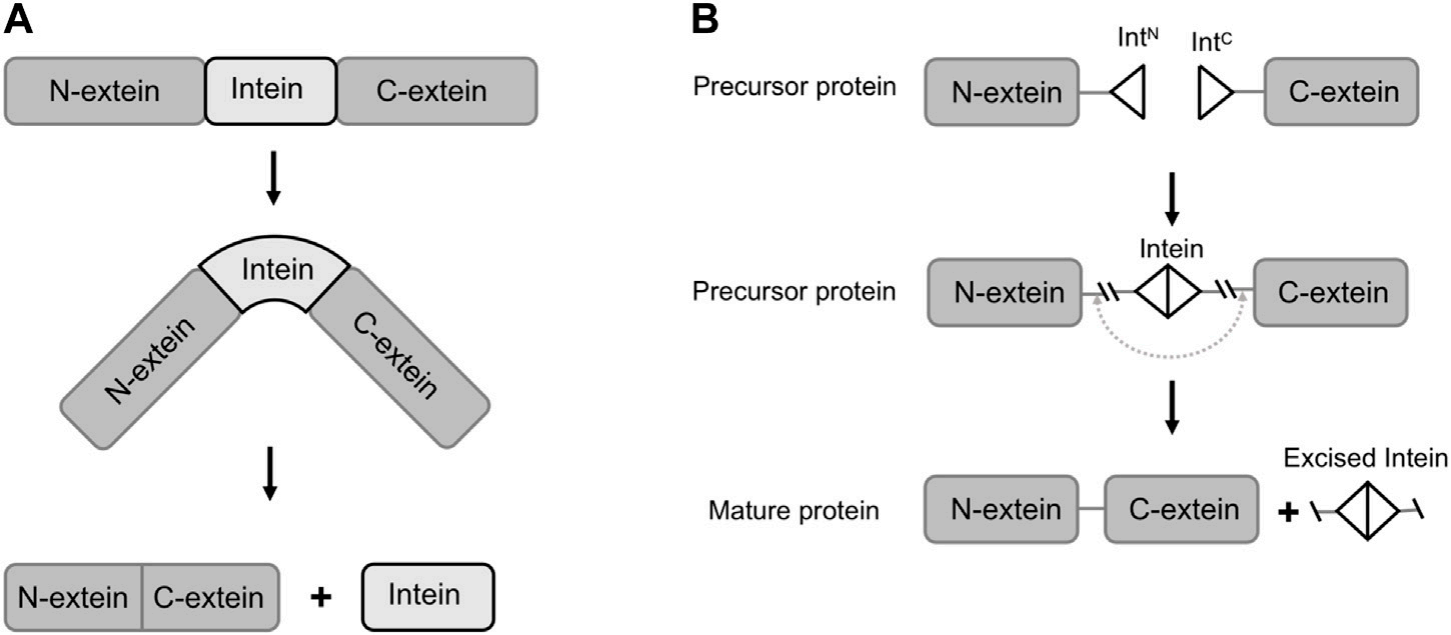
Schematic of protein splicing of inteins (*trans*-splicing and *cis*-splicing). **(A)** The translation of the precursor protein and its splicing in *cis*. **(B)** Split intein mediated protein splicing in *trans*. Int^N^, split N-intein; Int^C^, split C-intein.

Inteins are protein segments that are capable of ligating the flanking exteins (external proteins) into a new protein, a process known as protein splicing (Belfort et al., 2006). They are found in many natural organisms, such as bacteria, fungi and lower plants, and are usually embedded within essential proteins (Belfort et al., 2006). For example, Hirata *et al.* discovered the *Sce* VMA intein (vacuolar membrane ATPase subunit of *Saccharomyces cerevisiae*) in his study of ATPase from *Saccharomyces cerevisiae* by sequence alignment analysis in 1988 (Hirata et al., 1990). Naturally occurred inteins exist in several forms including full-length inteins, mini-inteins and naturally split inteins. The full-length inteins and mini-inteins are both *cis-*splicing inteins, with or without an endonuclease domain. Split inteins are *trans-*splicing inteins, with two fragments transcribed and translated by two independent genes. The *trans*-splicing requires the co-expression of both split intein fragments, namely N-intein (I_N_, fused with C-termini of an N-extein) and C-intein (I_C_, fused with N-termini of a C-extein). The split intein fragments subsequently associate to recover its activity and catalyze the ligation of N-extein and C-extein (Figure 1) (Stevens et al., 2016). In both *cis* or *trans*-splicing, the intein-mediated activities do not require assistance from any enzyme or co-factor, but only a proper folded structure of the expressed protein (Perler, 1998; Kwong et al., 2016). In the previous literature, the mechanisms and applications of inteins have been extensively reviewed (Wood et al., 1999; Mills et al., 2014). Nevertheless, with the rapid developments in synthetic biology research and technology, we have noticed that new and powerful tools are emerging to discover or evolve inteins with higher splicing efficacy. In the meantime, diverse and complex functions are achieved through engineered inteins mediated protein splicing. Therefore, in this paper, we first introduce the composition and function of naturally occurring inteins, and further review the recent developments and applications of inteins in synthetic biology.

## Naturally Occurred Intein: Composition and Splicing Mechanisms

In nature, protein splicing synthesizes two separate proteins (the inteins and exteins) under the control of a single gene by the precise excision of an internal protein segment and concomitantly ligation of the flanking regions (Topilina and Mills, 2014). Oftentimes these two separate proteins are both functional: the excised inteins contain homing endonucleases (HED) that can catalyze the lateral transfer of their DNA coding sequences by an intein homing mechanism, while the ligated exteins are mostly enzymes with specific functions (Chong and Xu, 1997; Perler, 2005; Cheriyan and Perler, 2009; Kwong et al., 2016). Previous research has categorized the inteins into three classes: class 1, class 2 and class 3 based on the mechanism of splicing from the extein. Here we mainly elaborate on the composition and splicing process of the class 1 inteins.

**FIGURE 2:**
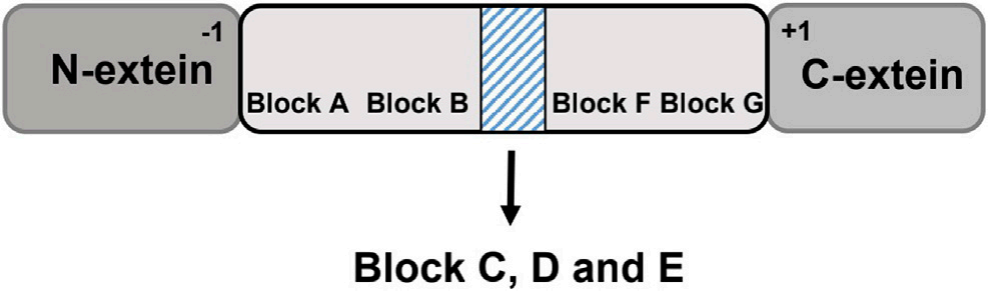
Conservative motifs of inteins facilitate protein splicing. The motifs A-G are identified in the intein domain. Block C, D and E are noted in the shadow box, where the split sites of naturally occurring split inteins and homing endonuclease domain (HED)are located.

Several amino acid residues and peptide sequences at certain positions of class 1 inteins are highly conservative (conservative motifs), and these conservative motifs are tightly related with the splicing reaction and splicing efficacy (Figure 2) (Chong and Xu, 1997). Shaorong *et al.* identified seven motifs (motif A-G) composed of a series of conserved amino acid residues. For example, motif A is pointed to as the first amino acid residue on the N-terminus of the intein for protein splicing, consisting of a hydroxyl- or thiol-containing residue (Ser, Thr, or Cys), while motif G is often found to be an Asn residue at the other junction site on the C-terminus with a His residue at the penultimate site beside Asn(Mills et al., 2014). Motif C and E are dodecapeptide regions acting as the homing endonucleases (Chong and Xu, 1997). These dodecapeptide motifs could recognize DNA and catalyze DNA cleavage. By doing so, the *Sce* VMA (Vacuolar Membrane ATPase in *Saccharomyces cerevisiae*) intein initiates a gene recombination process to disperse the intein genes to other strains (Perler, 1998).

**FIGURE 3:**
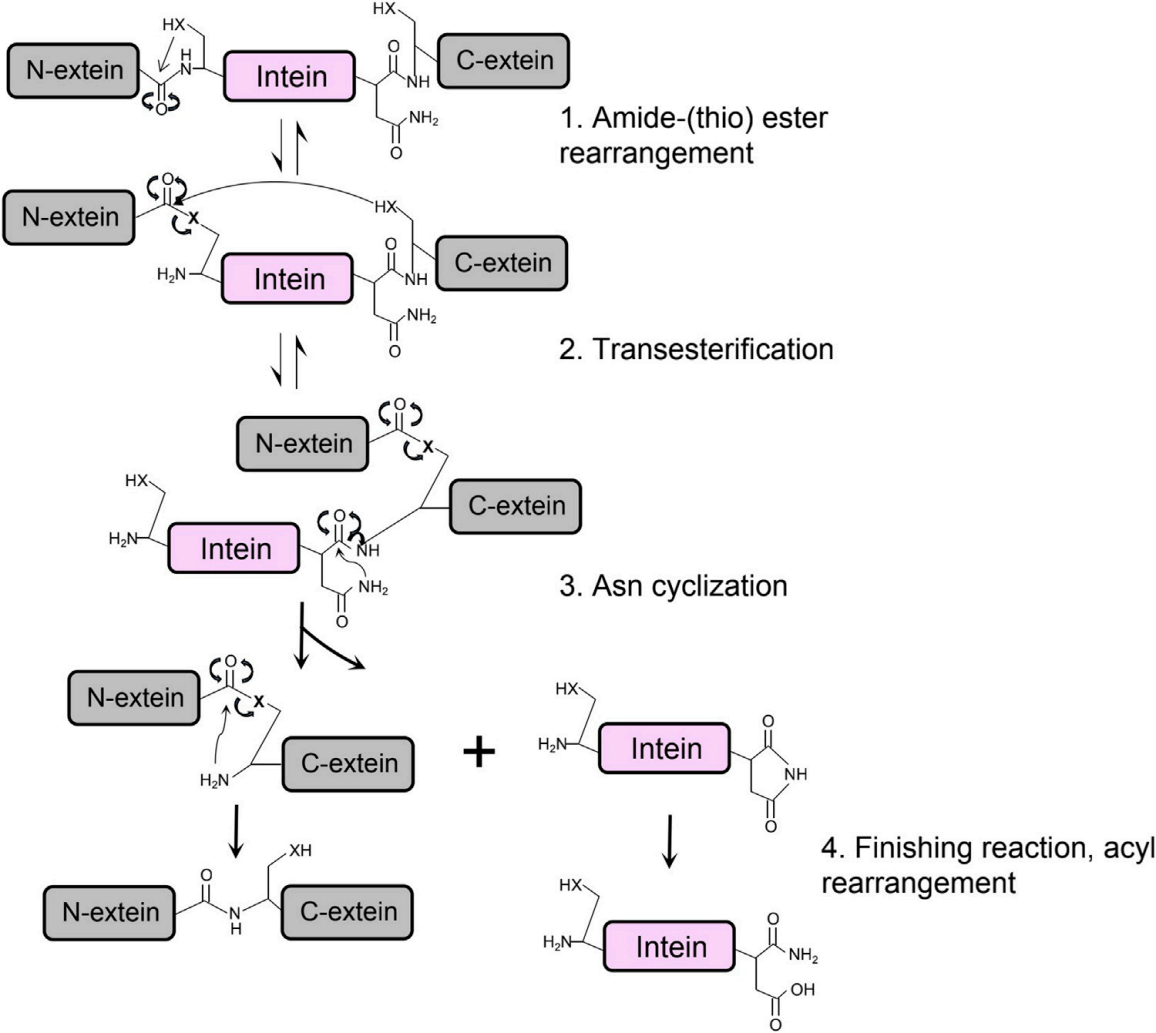
The protein splicing mechanism of class 1 intein composes four main steps, in which X is referred to an oxygen or sulfur atom.

The protein splicing of class 1 intein is achieved through structural conformational change and chemical bonds shifting on junction sites between intein and exteins, which can be summarized in four main steps (Figure 3) (Gorbalenya, 1998; Reitter and Mills, 2011). First, a nucleophilic attack by the N-terminal Cys or Ser of the intein converts the peptide bonding between the N-extein and intein to an ester or thioester group. Then, the transesterification transfers the N-extein from the side chain of the N-terminus of intein to the first residue of C-extein at the C-terminus of the intein, forming a branched intermediate. The Asn cyclization of the last amino acid residue on the C-terminus of intein frees the branched ester with a peptide bond cleavage, resulting in ligated exteins with an ester bond linkage. Finally, rapid conversion from the ester bond to the amide bond occurs to form the final ligated peptide (Southworth et al., 2000).

Some of the naturally occurring inteins (mini-intein) lack the endonuclease region and only conduct the protein splicing function (Chong and Xu, 1997; Telenti et al., 1997; Perler, 1998; Southworth et al., 2000). Telenti *et al.* reported the discovery of GyrA inteins retrieved from 7 mycobacterial species and subspecies. Especially, the *Mycobacterium xenopi* GyrA intein (*Mxe* GyrA) consisted of only 198 amino acid residues (AAs), as compared with the inteins with 420 AAs as analyzed from the rest six species. Sequence analysis confirmed the missing of the endonuclease region in *Mxe* GyrA (Telenti et al., 1997). Split inteins were also found in nature that can facilitate protein splicing in *tran*s (Figure 1) (Pinto et al., 2020). For instance, Evans *et al.* identified a naturally occurring split intein in the *dnaE* gene which encoded the catalytic subunit of DNA polymerase III in *Synechocystis sp.* PCC6803 (Evans et al., 2000). This DnaE split intein pairs contained the N-terminal half (123 AAs) and C-terminal half (36 AAs), encoded by two separate open reading frames in the genome of the original species (Evans et al., 2000). The two DnaE-intein fragments could be co-expressed in *Escherichia coli* (*E. coli*) and exhibit protein *trans*-splicing activity (Evans et al., 2000).

Besides the proximal amino acid residues on both sides of intein terminuses, splicing is also dependent on the reaction condition. Both *cis-* and *trans*-splicing could be optimized in certain conditions such as optimized pH or reducing environments (Perler and Adam, 2000; Shi et al., 2015; Zhang et al., 2015; Yu et al., 2016; Wang et al., 2018; Pinto et al., 2020; Kimura et al., 2021). For example, Zhang *et al.* found that a weakly acidic environment (pH ∼ 6.0–6.5) could facilitate the *Ssp* DnaB intein C-terminal cleavage activity (*cis*-splicing) (Zhang et al., 2015). Wang *et al.* reported that a basic environment (pH ∼ 8.5–9.5) benefitted the *Mxe* GyrA intein cleavage reactions (*cis*-splicing) (Wang et al., 2018). Recently, Pinto *et al.* pointed out that a weak basic condition (pH∼ 9.0) plus the minimal concentration of DTT (4 mM) promoted the splicing of the split inteins (*trans*-splicing) (Pinto et al., 2020).

## Engineering Intein by Synthetic Biology

In the last 20 years, synthetic biology has been bridging multiple disciplines to design and build novel biomolecular components, networks and pathways, and using these elements and knowledge to rewire and reprogram organisms (Khalil and Collins, 2010; Cameron et al., 2014; Singh, 2014; Mao et al., 2021; Tang et al., 2021). As nature’s escape artists, inteins can seamlessly stitch two proteins, leading to beneficial consequences such as the large protein assembly and the activation of certain factors without requiring assistance from any enzymes or co-factors (Ramsden et al., 2011; López-Igual et al., 2019). These features of intein hold great promise in addressing needs in biomanufacturing, sensing and diagnoses. However, the deficiency in the splicing efficacy and the lack of enough intein tools largely restrain the applications. Intein can proficiently excise itself from its natural host protein largely depending on the certain fixed junction sequences between exteins and inteins. Based on the current research, the splicing efficiency is often influenced by the proximal amino acid residues on both sides of intein terminuses, especially when recombinantly expressed in a non-native host (Wood et al., 1999; Pinto et al., 2020). These limitations hinder the applications and popularization of inteins since the successful splicing reaction experienced with one extein may not work on another one (Belfort et al., 2006; Pinto et al., 2020). In the past several years, researchers have successfully utilized strategies and tools from synthetic biology to evolve the inteins with enhanced splicing efficacy and expanded the current intein library.

Directed evolution has been proven to be a robust and reliable method to design and alter proteins towards the desirable biological functions, by generating random mutations in the target gene and imposing stringent selection conditions to identify proteins with optimized functionality. Multiple research groups have successfully used the directed evolution method to create genetic diversity of inteins and identified the ones with enhanced splicing efficiency (Wood et al., 1999; Marshall et al., 2015; Stevens et al., 2017). For example, Wood *et al.* conducted random mutagenesis on a mini-intein and coupled the intein activity to a selectable growth phenotype for screening. Specifically, they used *E. coli* deficient in cellular thymidylate synthase (TS) thus the resultant strain was unable to grow without thymine. They generated a pool of mini-inteins (containing the first 110 and the last 58 amino acids of the 441–amino acid *Mtu* RecA intein) by mutagenic PCR. To couple the splicing efficiency to the TS reporter system, intein–TS fusions were constructed in such a way that the active TS would be produced by intein-mediated splicing to rescue the host cells (Wood et al., 1999).

In 2017, Stevens *et al.* integrated both the rational design and directed evolution to engineer a naturally occurred split intein, and the improved version is capable of efficient splicing with tolerance on the local extein contexts. They chose one of the most commonly used split inteins *Npu* DnaE (*Nostoc punctiforme*, embedded within the catalytic subunit of DNA polymerase III), which already showed negligible sensitivity to the N-extein residues. However, *Npu* DnaE split intein has sequence preference to the catalytic cysteine (+1 position) and large hydrophobic residues (+2 position) of the C-extein. A previous study showed that these large hydrophobic residues were essential in maintaining the splicing rate due to a stabilizing effect between Phe_+2_ and His_125_ (a key catalytic residue in the last step of protein splicing, involving the cyclization of Asn_137_) (Sun et al., 2005; Shah et al., 2013). Less bulky +2 residues lead to a more dynamic His_125_ side chain with additional conformations that cannot catalyze the splicing. Therefore, they hypothesized that engineering the loop around His_125_ (residues 122–124) could potentially adjust the His_125_ conformational dynamics and therefore restrain the effect of the +2 residue on splicing kinetics. To implement this loop engineering, they conducted saturation mutagenesis on the His_125_ loop (residues 122–124) of *Npu* DnaE intein and coupled the splicing activity to antibiotic resistance in *E. coli* by reconstituting a split version of the aminoglycoside phosphotransferase (resistance to kanamycin) protein. Especially, this splicing was done in the presence of the unfavorable Gly_+2_. By this strategy, they selected mutants that showed remarkable kanamycin resistance with unfavorable C-extein +2 residue, indicating that the evolved intein could splice with minimal extein residues dependency (Stevens et al., 2017).

Besides evolving specific inteins for better performance, people also built intein bank to further explore the potential useful inteins. As mentioned previously, the splicing efficiency of inteins is largely dictated by the junction sequence between the inteins and exteins, and the deviation from the preferred junction sequence may lead to reduced splicing activity (Kwong and Wong, 2013). Therefore, an expanded library of sufficiently characterized inteins would enhance the probability of matching the target protein with a certain intein bearing a compatible junction sequence. Additionally, selecting split intein pairs that could work simultaneously and orthogonally with no cross-reactivity holds great promise to increase the splicing throughput and expand the application fields.

In 2020, Pinto *et al.* assessed 34 split inteins libraries and established a library of mutually orthogonal split inteins for both *in vivo* and *in vitro* applications. This study offered fully characterized and versatile toolboxes for scientists to choose based on the desired applications. They first chose 11 pairs of thoroughly characterized split inteins with high splicing reaction rates and efficiencies, and 3 inteins that were reported previously but not fully characterized (Perler, 2002; Dassa et al., 2009). To further expand the library, they extended their search to the intein database of viral and viral-like inteins assuming that these inteins would have faster splicing rates due to the short life cycle of the virus. They shortlisted the 50 inteins at the initials and screened down to 20 phylogenetically distant inteins, based on the assumption that homology is negatively correlated with orthogonality. With these stringent selection criteria, the total of 34 selected candidate inteins were unlikely to share a common ancestor intein since each of them had unique native exteins and shared low sequence homology. To assess the intein functionality and orthogonality, they developed a fast and indirect measurement of splicing activity reporter system, based on the intein-mediated reconstitution of fluorescent protein mCherry. They evaluated the *in vivo* or *in vitro* orthogonality of the split inteins and identified 15 mutually orthogonal pairs that could be used in diverse applications (Pinto et al., 2020).

Another challenge lied in identifying insertion sites of inteins. Besides sharing the similar difficulties in searching a general split site, the introduction of inteins brought an extra layer of complexity since the splicing efficiency largely depends on the extein junction sequences (Ho et al., 2021). To address this issue, Ho *et al.* used a mini-Mu transposon-based screening approach (intein-assisted bisection mapping (IBM) method) to reveal the split sites for a given protein. They first inserted a transposon randomly into a staging vector, which hosted a coding DNA sequence (CDS) of interest by an *in vitro* transposition reaction. The CDS with successful insertion was isolated by size selection and further ligated into a vector (Nadler et al., 2016; Zeng et al., 2018). The transposon was then substituted with a DNA fragment containing a split intein, the transcription and translation initiation elements for carboxyl-lobe expression and a selection marker. In-frame insertions with the correct orientation will thus split a CDS into two, in which the split intein fragments were fused with the amino-lobes (N-lobes) or carboxyl-lobe (C-lobes) under the separate control of two inducible promoters. The generated library was screened based on the selection rule that the clones displayed the function when both promoters were induced. The clones fulfilling the standard were then sequenced to reveal the split sites at the fusion joints. Using this method, they discovered clusters of split sites on five proteins (Calles and Lorenzo, 2013; Ho et al., 2021). Their work established a generalizable methodology to create split protein-intein fusions for synthetic biology.

## Applying Inteins in Synthetic Biology

As protein engineering tools, inteins have been widely used in protein purification, protein labeling, and protein cyclization. For example, inteins have been developed as self-cleavable linker during the purification to generate untagged protein (Zhang et al., 2015; Wang et al., 2018). Inteins were also applied in making recombinant C-terminal polypeptide α-thioesters, which were the crucial components for the semi-synthesis of chemically modified proteins using expressed protein ligation (EPL) (Flavell and Muir, 2009; Fong and Wood, 2010; Li, 2015; Sarmiento and Camarero, 2019). Protein *trans*-splicing mediated by split inteins (naturally-occurring or artificially designed) has been used in proteins modification, such as introducing the site-specific modification including phosphorylation, biotinylation, ubiquitination, glycosylation, and segmental isotopic labeling both *in vitro* and in cells (Qin et al., 2002; Rak et al., 2003; Shogren-Knaak et al., 2003; Chacko et al., 2004; Durek et al., 2004; Flavell and Muir, 2009; Borra and Camarero, 2017; Debelouchina and Muir, 2017). Inteins have also been utilized in generating cyclic proteins, in which the split intein fragments were fused to both sides of a target protein, and the N- and C-terminus of a target protein were joined through the association and splicing of the pairs (Topilina and Mills, 2014; Nanda et al., 2020). These applications have been well established and extensively reviewed. Apart from these works, we have noticed that the inteins have been playing important roles in multiple areas of synthetic biology ranging from biocomputing, living therapeutics to material assembly. Here, we further discuss these new efforts.

### Biocomputing

Split inteins are ideal tools for implementing digital logic. A protein can be divided into two and fused with a pair of split intein, such that the bipartite fragments remain individually inactive, and protein function is not restored until protein splicing occurs. In the work of Ho *et al*, after establishing a general workflow (IBM, as mentioned above) to identify the split sites for given proteins, they continued to demonstrate the universality of the method in engineering protein-based logic gates. They discovered multiple split sites within a repressor (tetracycline repressor, TetR) and an activator (the extracytoplasmic sigma factor 20, ECF20). TetR or ECF20 were reconstituted only when both the N-lobes and the C-lobes were present under the induction (induced by arabinose and DAPG (2,4-Diacetylphloroglucinol) respectively). The full protein either repressed or activated the expression of mScarlet (output), and effectively generated a NAND (TetR) or AND (ECF 20) logic (Ho et al., 2021).

**FIGURE 4:**
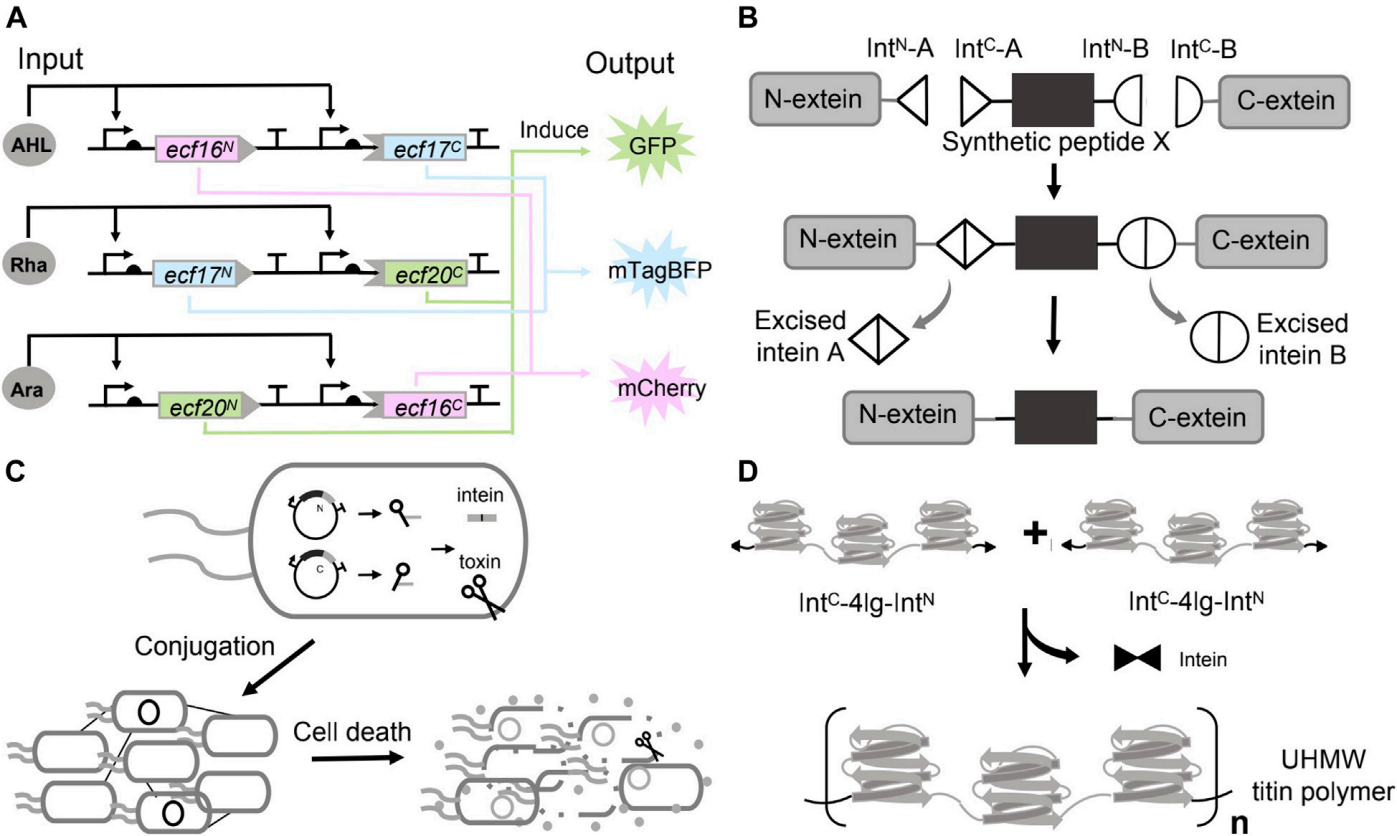
Use of inteins in synthetic biology. **(A)** Applying inteins to implement biocomputing. **(B)** Chemical modification of peptide or protein by inteins mediated protein splicing. **(C)** Living therapeutics constructed by the inteins directed splicing. **(D)** Materials assembly by protein splicing of inteins.

In another work, after establishing an extended library of orthogonal split inteins, Pinto *et al.* have further coupled the orthogonal split inteins with orthogonal split extracytoplasmic function (ECF) sigma factors to build modular logic AND gates that can be wired to build complex logic circuits. ECF sigma factors are the smallest and simplest alternative sigma factors. They split three orthogonal ECFs and fused each one with a pair of split intein (three pairs of split inteins are orthogonal to each other). The ECF proteins were reconstituted by the intein mediated *trans*-splicing. The fluorescence was detected only when both ECF halves were expressed, confirming the behavior of logic AND gates (Pinto et al., 2020). They have further connected the three AND gates to build a three-input three-output integrated logic circuit (Figure 4A). In each circuit, the promoter (induced by one inducer, and each inducer is defined as one input) drove the expression of a pair of unrelated intein split ECF halves. Therefore, the fluorescent output is only activated by having at least two inputs. The experimental results indicated that the design exhibited the expected logic behavior. These applications suggested the potentials of using orthogonal split inteins and split transcription factors to design complex cellular logic circuits.

### Generation of Semisynthetic Proteins

Chemical modification of proteins holds great potential for therapeutics engineering since it can help to understand the pharmacology and improve the property and effect of the drugs (Wold, 1981; Borra and Camarero, 2017). Incorporating non-canonical amino acids (ncAAs) in a site-specific manner can partially address the issue (Liu and Schultz, 2010). However, limitations exist such as the toxicity of ncAAs to the cells, and the difficulties in incorporating multiple ncAAs using the native transcription and translation machinery (Khoo et al., 2020). These problems could be potentially addressed by dividing the target proteins (with post-translational modification (PTM)) into multiple fragments, in which the fragments with the PTM were chemically synthesized, and stitching these parts back by inteins mediated splicing (Figure 4B) (Sarmiento and Camarero, 2019; Khoo et al., 2020). For example, Khoo *et al.* managed to synthesize the protein with PTM by *trans*-splicing in living eukaryotic cells, a method defined as tandem protein *trans*-splicing (tPTS) (Khoo et al., 2020). They divided the protein of interest (POI) into three fragments, namely the N-terminal, C-terminal parts and a central fragment (peptide X) containing the required modification (Nilsson et al., 2005). These three parts were ligated with two orthogonal split intein pairs (*Cfa* DnaE intein and *Ssp* DnaB). The protein N- and C-fragments were expressed by HEK cells, while the peptide X containing the ncAAs was generated by chemical synthesis and injected into the cells (Khoo et al., 2020). Their approach successfully inserted the synthetic peptide containing the homolysine or ornithine (ncAAs) at K71 into the P2X2 receptors (a trimeric ATP-gated ion channel that can be activated by ATP released during synaptic transmission), as validated by the protein function (Khoo et al., 2020).

### Living Therapeutics

Synthetic biology is paying attention to the rising field of living therapeutics. Instead of small molecules and protein drugs, researchers are developing genetically engineered cells as the basis for novel therapeutics. Inteins could endow the engineered cells with diverse and sophisticated functions, such as maintaining a dormant state (un-spliced) in the delivering host and activating to generate the therapeutics through the splicing at the target environment. In 2019, López-Igua *et al.* engineered *E. coli* carrying the split toxins constructs. The split toxins constructs were delivered through conjugation between the *E. coli* and the pathogen. The toxin–intein antimicrobial reagent was only activated in the pathogen that harbors specific transcription factors (Figure 4C). In their experimental design, they chose the toxic protein CcdB as the antimicrobial reagent since it locked up DNA gyrase with broken double-stranded DNA and ultimately caused cell death (Guérout et al., 2013). However, even the basal expression of a full-length toxin gene (*ccdB,* driven by the P_BAD_) was sufficient to kill the *E. coli* host. Consequently, they split the toxin gene by an intein (DnaE). The expression of the split toxin gene and reconstitution of the toxin could only be activated by ToxR, the essential transcription activator controlling the expression of cholera toxin, colonization factor and outer membrane protein of *V. cholerae*. By design, the intein-assisted method enabled targeted killing of pathogenic bacteria without harming beneficial members of host-microbiota (Van Melderen et al., 1996).

As another example, inteins could assemble the therapeutics which were too large to deliver in the gene therapy. CRISPR/Cas9 provides a possible solution to target and edit virtually any gene to remove the diseases. A major obstacle, however, is that the size of Cas9 (>4 kb) impedes its efficient delivery. Truong *et al.* reported the use of a split intein mediated Cas9 system in a dual-vector recombinant adeno-associated virus (rAAV) system, in which rAAV invaded cells, delivered the split-Cas9 elements and reconstituted Cas9 via protein splicing of the *Npu* DnaE split intein (Truong et al., 2015).

As another example, the adeno-associated viral (AAV) vector-based gene therapy biologics can cure an inherited form of blindness (Tornabene et al., 2019). Again, the limited cargo capacity of the AAV vector inhibits its potential use (Tornabene et al., 2019). Tornabene *et al.* proved that this problem in retinal gene therapy could be ameliorated by a split intein assisted assembly strategy. In their experiments, they successfully delivered multiple AAV vectors, each encoding one of the fragments of target proteins flanked by the split intein, and reconstituted the large ATP binding cassette subfamily A member 4 (ABCA4) as well as centrosomal protein 290 (CEP290) in the retina of mice, pigs and in human retinal organoids to cure the inherited retinal diseases (Tornabene et al., 2019).

### Materials Assembly

Artificial high-performance polymer materials bear unique properties such as high-strength and have broad application prospects. However, these materials are mostly derived from petroleum and are non-degradable and non-sustainable (Wegst et al., 2015; Yang et al., 2017). Synthetic biology has engineered microorganisms to produce a wide range of degradable biomaterials (Chen et al., 2014; Gilbert et al., 2021; Tang et al., 2021). Many natural materials with high mechanical properties are hierarchically assembled ultra-high molecular weight (UHMW) proteins that have highly repetitive amino acid sequences. Nevertheless, these UHMW repetitive proteins were extremely difficult to produce in microorganisms due to the genetic instability, low transformation efficiency and metabolic burden (Tang and Chilkoti, 2016; Rugbjerg et al., 2018). In 2019, Bowen *et al.* showed that the problem could be addressed through *in vivo* protein polymerization catalyzed by split inteins. They designed the monomer construct that contained the 10 repeats of a *Nephila clavipes* MaSp1 dragline spidroin consensus sequence and flanked by a pair of complementary, fast reacting split inteins in the form of Int^C^-monomer-Int^N^, where Int^C^ and Int^N^ represent the C- and N-half of the split inteins, respectively. Expression of this monomer alone, however, caused the protein cyclization instead of polymerization, probably due to the structural flexibility of the monomer permitted the joint of the N- and C-termini. To prevent intramolecular cyclization, they have devised a seed chain polymerization (SCP) method by first inducing a “seed protein”, which contained only one reactive Int^N^ domain fused at the C-terminus of the seed. After a certain time, the IntC-monomer-IntN cassette was subsequently expressed. Considering both the ligation kinetics and the protein synthesis rate, the Int^C^ domain at the N-terminus of the monomer should react with a seed or linear chain before its C-terminal Int^N^ domain can be translated, resulting in linear intermolecular ligation instead of cyclization. Using this method, they have successfully synthesized a spider silk protein with a molecular weight of 300 kDa in *E. coli* (Bowen et al., 2019).

Recently, Bowen *et al.* has further used a similar strategy to fabricate megadalton muscle titin polymers (Figure 4D). They fused the C- and N-terminal halves of a split intein to the N- and C-termini of a short titin subunit containing four Ig domains. The expression of monomers spontaneously initiated the splicing reactions and covalently linked the monomers to form the UHMW proteins (20% of the purified titin polymers have a molecular weight over 5 MDa). In this case, the subunit of four Ig domains was rigid to prevent the cyclization. They next processed these UHMW proteins into macroscale monofilament fibers, and showed that these high-performance fibers exhibited high strength, toughness, and damping energy (Bowen et al., 2021).

## Summary and Perspective

Over the last 3 decades, research about inteins is progressively pushing the limits from identification and characterization of inteins to productive utilization. Recently, the concepts and tools from synthetic biology have been further integrated with inteins to enable new and diverse functions. In this review, we have introduced several inteins mediated applications ranging from living therapeutics engineering to material assembly. Obviously, inteins can empower the system with more flexibility and functionality. Previously research groups have utilized SpyTag/SpyCatcher system in the field of engineered living material. For example, they have decorated the polypeptides with either SpyTags or SpyCatchers, which can react to polymerize to form the protein polymer (Dai et al., 2021). The role of the SpyTag/SpyCatcher could be replaced with the split intein pairs, which can stitch the peptide seamlessly. It is worthy to note that the library of the orthogonal split intein pairs can link monomers with high selectivity. Therefore, different polypeptides could be assembled in a certain sequence to form the “block polymer”. Inteins could further be coupled with tools of optogenetics, such that the living therapeutics could be only activated via light-controlled intein mediated splicing.

Limitations still exist. For example, splitting the target protein often leads to the misfolding of the protein and the formation of inclusion bodies. Although new inteins are consistently discovered, the number of feasible and reliable tools and orthogonal pairs (split inteins) are still deficient. These problems could be possibly improved with protein design by deep learning and large-scale screening by robotic-driven automation. With the advancement and enrichment in the inteins, new genetic parts and networks, we are expecting to see more exciting and creative roles played by inteins in versatile applications.
